# [^68^Ga]Ga-NODAGA-E[(cRGDyK)]_2_ PET and hyperpolarized [1-^13^C] pyruvate MRSI (hyperPET) in canine cancer patients: simultaneous imaging of angiogenesis and the Warburg effect

**DOI:** 10.1007/s00259-020-04881-0

**Published:** 2020-07-03

**Authors:** Andreas Clemmensen, Adam E Hansen, Pernille Holst, Christina Schøier, Sissel Bisgaard, Helle H Johannesen, Jan Henrik Ardenkjær-Larsen, Annemarie T Kristensen, Andreas Kjaer

**Affiliations:** 1 Department of Clinical Physiology, Nuclear Medicine & PET and Cluster for Molecular Imaging, Department of Biomedical Sciences, Rigshospitalet and University of Copenhagen Denmark, Copenhagen, Denmark; 2 Department of Veterinary Clinical Sciences, Faculty of Health and Medical Sciences, University of Copenhagen, Frederiksberg, Denmark; 3 Department of Electrical Engineering, Technical University of Denmark, Kongens Lyngby, Denmark

**Keywords:** Positron emission tomography, Hyperpolarized magnetic resonance spectroscopy imaging, [1-^13^C]pyruvate, Angiogenesis-PET, ^68^Ga-RDG, Cancer, hyperPET, Glycolysis

## Abstract

**Purpose:**

Cancer has a multitude of phenotypic expressions and identifying these are important for correct diagnosis and treatment selection. Clinical molecular imaging such as positron emission tomography can access several of these hallmarks of cancer non-invasively. Recently, hyperpolarized magnetic resonance spectroscopy with [1-^13^C] pyruvate has shown great potential to probe metabolic pathways. Here, we investigate simultaneous dual modality clinical molecular imaging of angiogenesis and deregulated energy metabolism in canine cancer patients.

**Methods:**

Canine cancer patients (*n* = 11) underwent simultaneous [^68^Ga]Ga-NODAGA-E[(cRGDyK)]_2_ (RGD) PET and hyperpolarized [1-^13^C]pyruvate-MRSI (hyperPET). Standardized uptake values and [1-^13^C]lactate to total ^13^C ratio were quantified and compared generally and voxel-wise.

**Results:**

Ten out of 11 patients showed clear tumor uptake of [^68^Ga]Ga-NODAGA-RGD at both 20 and 60 min after injection, with an average SUV_mean_ of 1.36 ± 0.23 g/mL and 1.13 ± 0.21 g/mL, respectively. A similar pattern was seen for SUV_max_ values, which were 2.74 ± 0.41 g/mL and 2.37 ± 0.45 g/mL. The [1-^13^C]lactate generation followed patterns previously reported. We found no obvious pattern or consistent correlation between the two modalities. Voxel-wise tumor values of RGD uptake and lactate generation analysis revealed a tendency for each canine cancer patient to cluster in separated groups.

**Conclusion:**

We demonstrated combined imaging of [^68^Ga]Ga-NODAGA-RGD-PET for angiogenesis and hyperpolarized [1-^13^C]pyruvate-MRSI for probing energy metabolism. The results suggest that [^68^Ga]Ga-NODAGA-RGD-PET and [1-^13^C]pyruvate-MRSI may provide complementary information, indicating that hyperPET imaging of angiogenesis and energy metabolism is able to aid in cancer phenotyping, leading to improved therapy planning.

## Introduction

Cancer is responsible for more than one-third of years lost, and the rate is rising [1]. Cancer covers a multitude of mutations, each a disease in itself, and in an effort to generalize the common traits, Hanahan and Weinberg formulated ten hallmarks of cancer [2]. Several of the hallmarks can be accessed by clinical molecular imaging, such as positron emission tomography (PET). One of these is deregulated cellular energetics, also simply known as the Warburg effect [3], which is routinely exploited in the clinic by PET with the glucose analog 2-[^18^F]fluoro-2-deoxy-d-glucose ([^18^F]FDG) to diagnose and stage cancer disease. Other hallmarks can be assessed using other PET tracers, such as 3-deoxy-3-[^18^F]fluorothymidine ([^18^F]FLT) for proliferation and [^68^Ga]Ga-NODAGA-E[(cRGDyK)]_2_ (RGD) for angiogenesis.

Hyperpolarized ^13^C magnetic resonance spectroscopic imaging (MRSI) is a non-invasive molecular imaging modality, which is currently entering clinical use [4–7]. Here, the technique of dissolution dynamic nuclear polarization (dDNP) overcomes the inherently low sensitivity of MRI by increasing the ^13^C signal by 5 orders of magnitude [8]. Hyperpolarized ^13^C-MRSI with [1-^13^C]pyruvate can be utilized to interrogate the Warburg effect, where the deregulated cellular energetics with an elevated aerobic glycolysis is reflected by the appearance of [1-^13^C]lactate [9–14]. Integrated PET/MRI allows for simultaneous [^18^F]FDG-PET and hyperpolarized [1-^13^C]pyruvate-MRS in the hyperPET setup [15, 16]. Comparison studies show a general spatial concurrence between patterns of FDG uptake and lactate generation [17, 18], as well as a correlation between values of [^18^F]FDG uptake and lactate generation [19, 20], but with an apparent tumor type dependence, which we hypothesized may allow for a more specific metabolic profiling of the deregulated cellular energetics using hyperPET [20].

In the present work, we expand the hyperPET approach to include imaging of another key hallmark of cancer, *angiogenesis*. During cell proliferation, the surrounding extra-cellular matrix (ECM) is remodeled, and in this process, integrins are essential cell adhesion receptors [21, 22]. One subtype, integrin α_V_β_3_, is expressed in newly formed endothelial cells of the vasculature, and the experimental, in-house developed, PET tracer [^68^Ga]Ga-NODAGA-E[(cRGDyK)]_2_ (RGD) binds to this receptor [23–25]. Integrin α_v_β_3_ is overexpressed in several types of cancer [26], and identifying these can lead to improved treatment, e.g., higher predictability of anti-angiogenic therapy. Imaging of angiogenesis using RGD as the integrin α_V_β_3_ antagonist first started with [^18^F]Galacto-RGD [27, 28]. With the introduction of the ^68^Ge/^68^Ga generator, chelation chemistry enabled fast and relatively stable labelling. Several chelators have been used [29–32], with dimeric NODAGA emerging as an attractive compromise between affinity and stability [33, 34]. Combined with the relatively short half-life of ^68^Ga, these factors make an evaluation of angiogenesis by temporal uptake of [^68^Ga]Ga-NODAGA-RGD relevant.

While [^68^Ga]Ga-NODAGA-RGD-PET and hyperpolarized [1-^13^C]pyruvate-MRSI have been extensively examined and validated individually [24, 33, 35–37], the two molecular imaging modalities have not been compared in a realistic, clinically relevant model. This hyperPET combination could, thus, provide simultaneous clinical imaging of angiogenesis and tumor energy metabolism and be utilized to investigate a possible relationship between those hallmarks of cancer as well as selection of treatment regime. In cancerous hypoxic tissue, angiogenesis is induced, and cells will switch to anaerobic metabolism, leading to the basic hypothesis that the [^68^Ga]Ga-NODAGA-RGD uptake and [1-^13^C]lactate generation are correlated.

Here, we investigate the combined molecular imaging of angiogenesis by [^68^Ga]Ga-NODAGA-RGD-PET and deregulated energetics by hyperpolarized [1-^13^C]pyruvate MRSI in canine patients with spontaneous cancers. The overall aim was to evaluate whether hyperPET can evaluate angiogenesis and tumor metabolism simultaneously in canine cancer patients prior to human clinical use. More specifically, we compare general tumor angiogenesis as demonstrated by [^68^Ga]Ga-NODAGA-RGD uptake with lactate generation, including patterns of tumor uptake heterogeneity between the modalities.

## Materials and methods

### Canine cancer patients

All procedures were approved by the Ethics and Administrative Committee, Department of Veterinary Clinical Sciences, Faculty of Health and Medical Sciences, University of Copenhagen. Canine cancer patients (*n* = 11) were included in the study after solid cancer had been verified by histopathology and where advanced tumor imaging could provide important information affecting the further treatment. On the day of examination, the patient received an intramuscular injection of methadone (Comfortan, 0.2 mg/kg) and two intravenous catheters were placed on separate extremities by experienced clinical veterinary staff. Upon arrival to the imaging facility, the canine cancer patient received an intravenous injection of diazepam (DAK, 0.5 mg/kg). Anesthesia was induced with a bolus injection of propofol (PropoVet, 4 mg/kg); the patient was intubated, connected to a ventilator, and supplied with oxygen and 2–3% Sevoflurane. The heart rate, oxygen saturation, and blood pressure were monitored throughout the procedure.

### [1-^13^C]pyruvate sample preparation

[1-^13^C]pyruvic acid (Sigma Aldrich, Denmark) was mixed with an electron paramagnetic agent (AH111501, Syncom) to a concentration of 15 mM. One milliliter of this solution was hyperpolarized in a 5 T SpinLab clinical polarizer (GE Healthcare, Denmark) for approximately 2 h, before being dissolved in 40 mL 0.1 mM EDTA dissolution media and neutralized in 14.6 mL buffer to a final concentration of 250 mM [1-^13^C]pyruvate.

### [^68^Ga]Ga-NODAGA-E[(cRGDyK)]_2_ synthesis

Labeling of NODAGA-E[(cRGDyK)]_2_ (ABX GmbH) was performed using a Modular-Lab PharmTracer (Eckert & Ziegler). The ^68^Ge/^68^Ga generator (IGG100; Eckert & Ziegler) was eluted with 6 mL of 0.1 M HCl. The eluate was concentrated on a Strata-XC cartridge and eluted with 700 μL of NaCl/HCl. Then, 30 nmol of NODAGA-E[(cRGDyK)]_2_ dissolved in 50 μL of TraceSelect water was labeled in 1000 μL of 1.4 M NaOAc buffer pH 4.5, and 400 μL of 50% EtOH at 60 °C for 5 min. The final product, [^68^Ga]Ga-NODAGA-E[(cRGDyK)]_2_, was purified on a SepPak tC2 plus cartridge (Waters), eluted with 50% ethanol, and formulated with isotonic saline to a total volume of 9 mL. The resulting yield was 501 ± 106 MBq of [^68^Ga]Ga-NODAGA-E[(cRGDyK)]_2_ for each production with a radiochemical purity ≥ 98%.

For quality control analysis, a high-performance liquid chromatograph (Ultimate 3000; Dionex) was performed on a Kinetex C18 column (2.6 μm, 100 Å, 50 × 4.6 mm; Phenomenex) and with the UV and radio-detector connected in series. The mobile phases were eluent A, 0.1% trifluoroacetic acid in H_2_O, and eluent B, 0.1% trifluoroacetic acid in MeCN. For thin-layer chromatography, a ScanRam scanner and iTLC-SG plates (Agilent Technologies) were used. The mobile phase was 77 g/L ammonium acetate in water/methanol (1:1). For gas chromatography, a Shimadzu GC2014 was used with a Zebron ZB-WAX 30 m × 0.53 mm × 1.00 μm column.

### Imaging protocol

All imaging was acquired using a combined clinical PET/MR scanner (mMR Biograph, Siemens) and a dual-tuned ^1^H/^13^C transmit/receive flex surface coil (RAPID Biomedical, Germany) placed over the primary tumor.

Localizer images were followed by relevant anatomical ^1^H T1- (TSE; TR 4000 ms, TE 89 ms, voxel size, 0.6 × 0.5 × 3.0 mm; 19 slices) and T2- (TSE; TR 550 ms, TE 6.5 ms, voxel size 0.7 × 0.6 × 3.0 mm; 27 slices) weighted MR imaging.

A single-bed, 5 min PET acquisition was performed 20 and 60 min after intravenous injection of 8.0 MBq/kg_BW_ of [^68^Ga]Ga-NODAGA-E[(cRGDyK)]_2_, corresponding to 0.713 ± 0.19 μg/kg_BW_ peptide. Between the two PET time points, ^13^C-MRSI was performed. The primary sequence was a ^13^C CSI acquired 30–45 s after injection of 0.58 mL/kg_BW_ hyperpolarized [1-^13^C]pyruvate solution (CSI; TR 80 ms, flip angle 10°, bandwidth 10 kHz, FOV 80–160 mm, slice thickness 13 mm, matrix 16 × 16 [circular truncation], acquisition time 11 s). The field of view was adjusted according to the organ of interest. After all other imaging sequences was completed, a transverse fat-saturated T1-weighted TSE with the same parameters as the first T1 TSE was performed 4–5 min after 0.1 mL/kg_BW_ gadolinium injection (Gadovist) in seven of the patients.

### PET reconstruction

[^68^Ga]Ga-NODAGA-RGD-PET was reconstructed using vendor supplied OP-OSEM3D with 4 iterations and 21 subsets into a 344 × 344 matrix size and with a 4 mm Gaussian filter applied. Due to the limited sensitivity profile of the MR surface coil used, the MR-generated 511 keV attenuation maps were inadequate for correct PET quantification and were manually corrected based on the body outline in the uncorrected PET image [38]. The attenuation map correction was performed using the MINC toolbox software [39] and returned to the original DICOM container for correct reconstruction. The correction method was validated by comparing corrected attenuation maps to complete attenuation maps made using the scanner body coil, which was available for some patients only.

### Image quantification

Regions of interest (ROI) were delineated on anatomical MRI, primarily the contrast-enhanced, fat-saturated T1-weighted TSE, by an experienced radiologist. It was attempted to avoid vessels and major areas of necrosis, before copying the ROI to functional imaging.

### ^13^C-MRSI quantification

The area of [1-^13^C]pyruvate, [1-^13^C]lactate, [1-^13^C]alanine, and [1-^13^C]pyruvate hydrate peaks were quantified using a general linear model implemented in Matlab (The MathWorks) and applied in the time domain. When reporting a metabolite ratio, the normalization was performed to the sum of all modeled peak areas. For visualization of the [1-^13^C]lactate to total ^13^C ratio, voxels which had a pyruvate peak height larger than five times the standard deviation of the noise in a background region of the spectrum were used and interpolated to the anatomical MRI resolution.

### Voxel-wise comparison of PET and CSI

For the purpose of a voxel-wise comparison, PET images (voxel size 2.1 × 2.1 × 2.0 mm^3^) were resampled to the MRSI (voxel size 5.0–7.5 × 5.0–10.0 × 13 mm^3^). Resampling was performed using scripts based on MINC [39]. The voxel-wise comparison was carried out for MRSI voxels with at least 90% of their volume within the radiologist-defined ROI.

### Statistics

All statistical tests were performed in Prism (version 8.3, Graphpad, La Jolla), and results presented with standard error of the mean. All comparisons were performed using standard parametric, two-tailed *t* tests. Values of *p* < 0.05 were considered statistically significant.

## Results

**Fig. 1:**
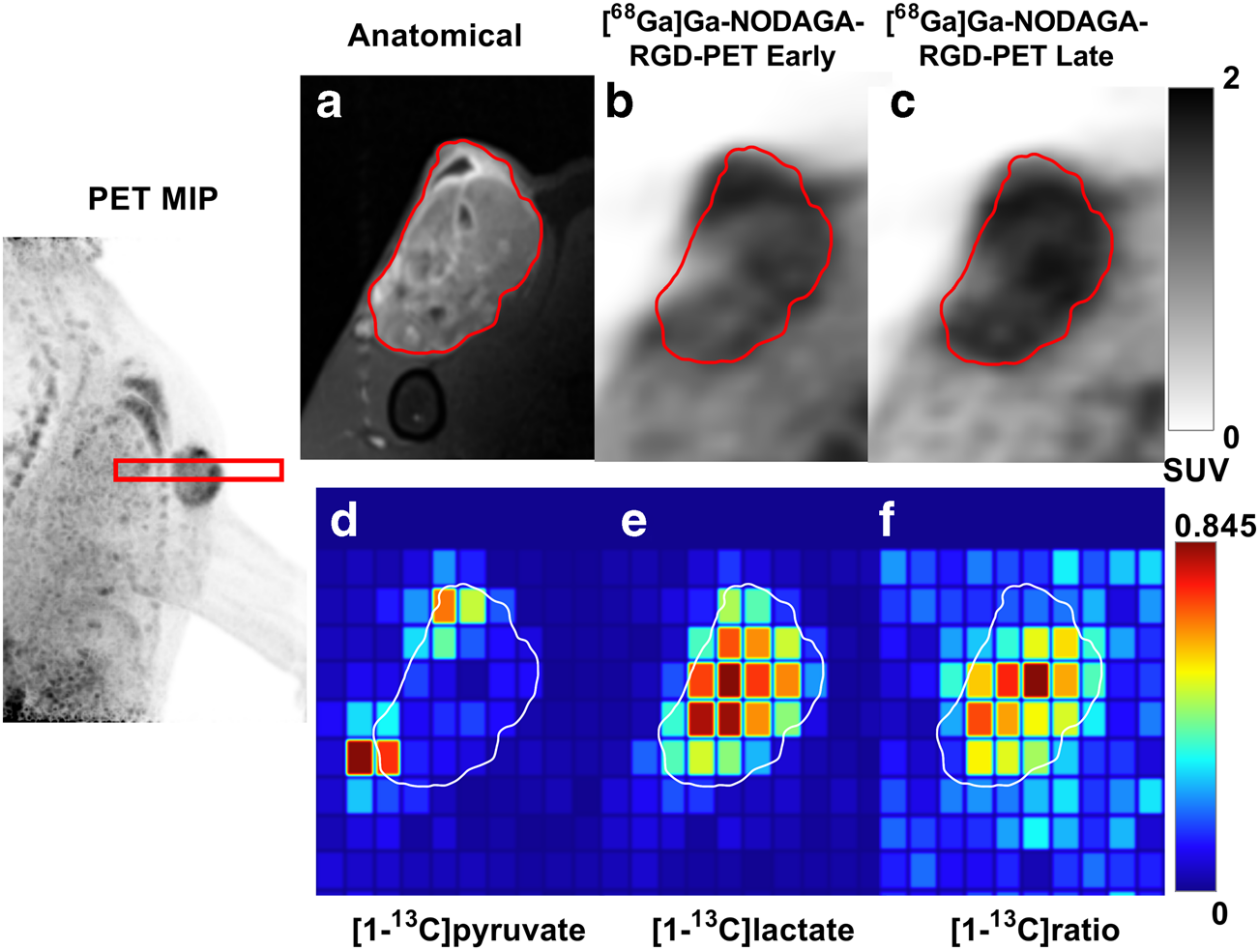
Example of hyperPET images from patient #1 with a soft tissue sarcoma adjacent to humerus. Far left is an RGD-PET MIP showing the tumor location and ^13^C-MRSI slice (red box). Panels **a**–**f** show imaging in the plane of the ^13^C-MRSI. Gd-enhanced T1-weighted anatomical MRI with tumor region of interest (ROI) outlined in red (**a**), along with [^68^Ga]Ga-NODAGA-RGD PET uptake images at 7 (**b**) and 53 min (**c**). Panels **d**–**f** show false-color images of metabolite distribution for [1-^13^C]pyruvate (**d**), for [1-^13^C]lactate (**e**), and ratio of [1-^13^C]lactate to total ^13^C (**f**) signal as determined by ^13^C-MRSI. The ^13^C-MRSI is shown in the acquired resolution

Eleven canine cancer patients were included. All patients underwent [^68^Ga]Ga-NODAGA-RGD PET at two timepoints (18 ± 1 min, range [7; 22] and 60 ± 2 min, range [53; 67]) and seven of these were also imaged using hyperpolarized [1-^13^C]pyruvate MRSI. For one patient, the exam had to be aborted prior to the MRSI procedure due to anesthetic complications, and for the remaining three patients, the polarizer was unavailable due to maintenance or technical failure. An overview of patient characteristics can be found in Table 1. Figure 1 shows an example of the hyperPET exam for patient 1.

**Table 1:** Overview over canine patients included in this study, their tumor types, and locations

ID	Weight (kg)	Tumor type	Tumor location	Imaging
1	26	Soft tissue sarcoma	Humerus	[^68^Ga]Ga-NODAGA-RGD-PET + [1-^13^C]pyruvate CSI
2	34	Malignant melanoma	Ungual	[^68^Ga]Ga-NODAGA-RGD-PET + [1-^13^C]pyruvate CSI
3	41	Osteosarcoma	Femur	[^68^Ga]Ga-NODAGA-RGD-PET + [1-^13^C]pyruvate CSI
4	27	Ectopic follicular thyroid carcinoma with osseous metaplasia	Basihyoid	[^68^Ga]Ga-NODAGA-RGD-PET + [1-^13^C]pyruvate CSI
5	28	Soft tissue sarcoma	Humerus	[^68^Ga]Ga-NODAGA-RGD-PET + [1-^13^C]pyruvate CSI
6	30	Thyroid carcinoma	Gl. thyroidea	[^68^Ga]Ga-NODAGA-RGD-PET + [1-^13^C]pyruvate CSI
7	34	Thyroid carcinoma	Gl. thyroidea	[^68^Ga]Ga-NODAGA-RGD-PET + [1-^13^C]pyruvate CSI
8	43	Thyroid carcinoma	Gl. thyroidea	[^68^Ga]Ga-NODAGA-RGD-PET
9	32	Chondrosarcoma	Ulna	[^68^Ga]Ga-NODAGA-RGD-PET
10	21	Thyoid carcinoma	Gl. thyroidea	[^68^Ga]Ga-NODAGA-RGD-PET
11	22	Ectopic follicular thyroid carcinoma	Regio laryngea	[^68^Ga]Ga-NODAGA-RGD-PET

### [^68^Ga]Ga-NODAGA-RGD-PET tumor uptake shows little time dependence

**Fig. 2:**
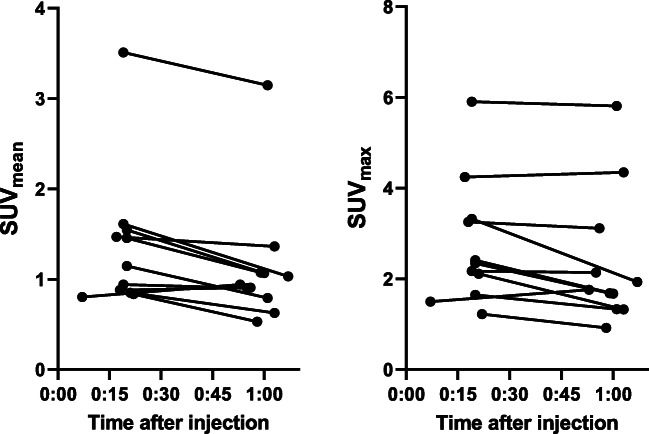
SUV_mean_ and SUV_max_ values of [^68^Ga]Ga-NODAGA-E[(cRGDyK)]_2_-PET at 20- and 60-min post injection

Patients (*n* = 11) showed varying degrees of [^68^Ga]Ga-NODAGA-RGD PET uptake in tumors, with an average SUV_mean_ of 1.36 ± 0.23 g/mL and 1.13 ± 0.21 g/mL at early and late imaging time points, respectively (Fig. 2). A similar pattern was seen for SUV_max_ values, which were 2.74 ± 0.41 g/mL and 2.37 ± 0.45 g/mL. The changes correspond to a drop of 16.1% (*p* = 0.012, paired t-test) for SUV_mean_ and 12.4% (*p* = 0.056, paired t-test) for SUV_max_.

### [^68^Ga]Ga-NODAGA-RGD uptake and ^13^C-lactate ratio in tumors across patients are not related

**Fig. 3:**
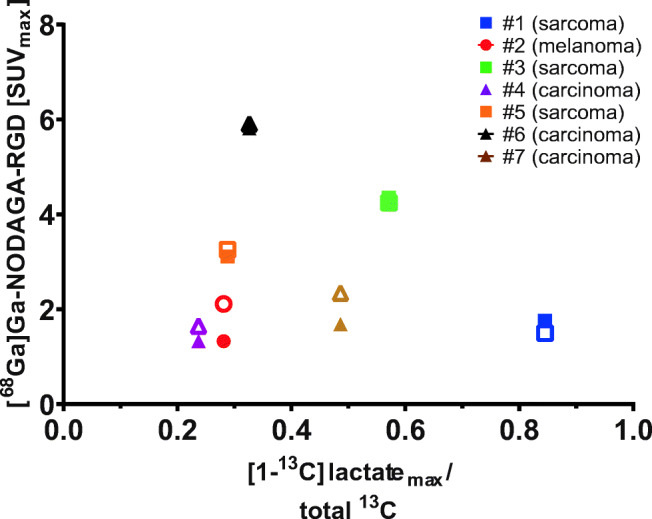
Scatter plot of maximum [1-^13^C]lactate to total ^13^C ratio values and maximum [^68^Ga]Ga-NODAGA-RGD-PET [SUV] at early (hollow) and late (filled) time points. Legend refers to patient numbers given in Table 1

A scatter plot of maximum [^68^Ga]Ga-NODAGA-RGD tumor uptake and lactate generation across patients is shown in Fig. 3. Tumors show a wide range of both RGD uptake (SUV 1–6) and lactate ratio (0.2–0.8). We found no direct pattern or correlation between the two modalities across patients, or across cancer types as was shown by Hansen et al. [20].

### Both [^68^Ga]Ga-NODAGA-RGD PET and ^13^C-pyruvate MRSI show heterogeneous tumor uptake

**Fig. 4:**
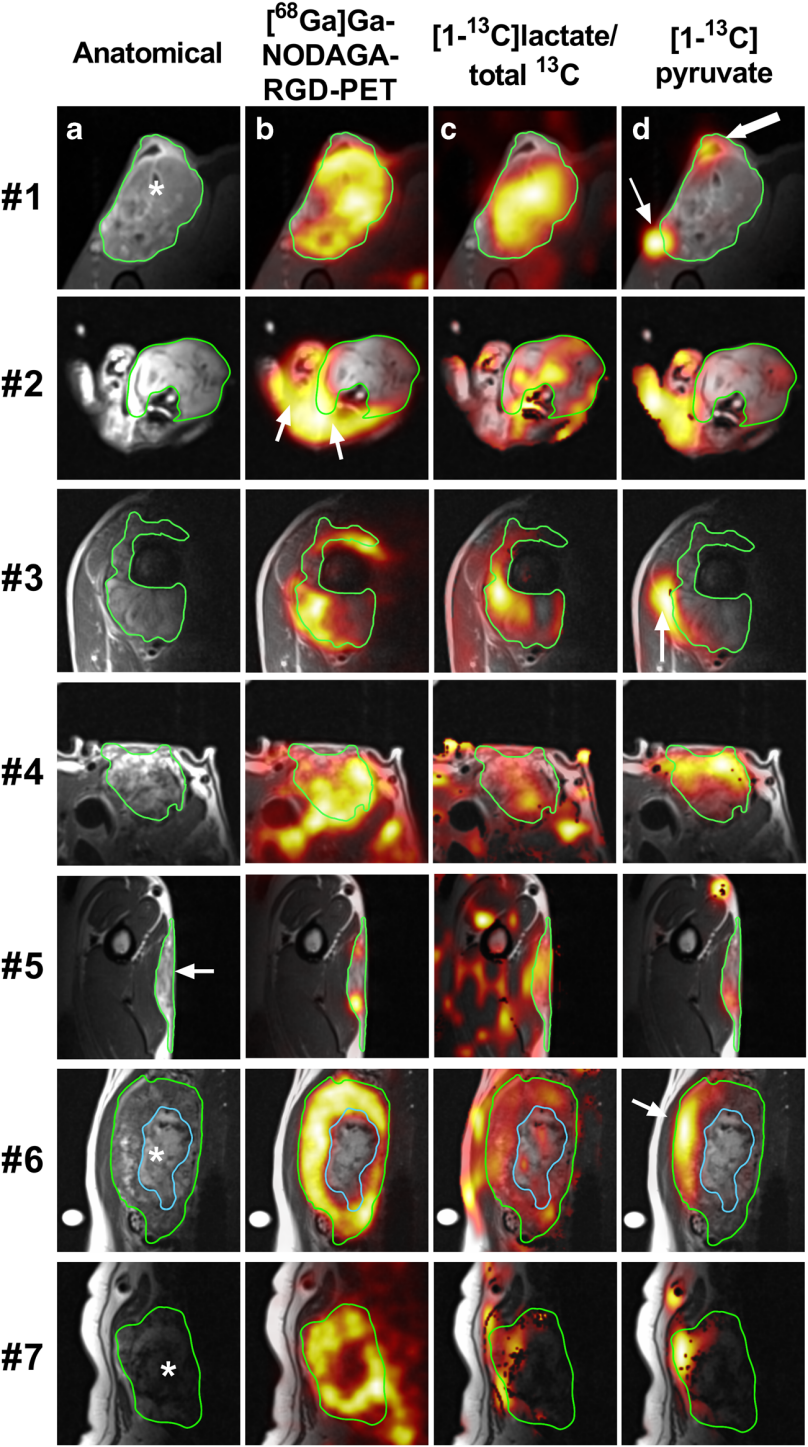
Comparison of late (50/60 min) [^68^Ga]Ga-NODAGA-RGD-PET distribution and [1-^13^C]lactate to total ^13^C ratio (color overlays) for seven patients. Tumor delineation is illustrated in green, and white arrows refer to details described in the Results section of the main text

Figure 4 shows spectroscopic imaging of lactate to total ^13^C ratio from ^13^C MRSI together with [^68^Ga]Ga-NODAGA-RGD PET for all seven complete hyperPET examinations. Summarizing the trends overall, both lactate ratio and [^68^Ga]Ga-NODAGA-RGD PET uptake display a heterogeneous pattern of enhancement in tumors. The individual patients are discussed in more detail below.

The tumor of patient #1 (soft tissue sarcoma) generally has high values for both [1-^13^C]lactate to total ^13^C ratio and [^68^Ga]Ga-NODAGA-RGD uptake, both primarily confined to the tumor. The tumor appears anatomically heterogeneous, possibly with very small areas of necrosis (white star, Fig. 4(1a)). Also, intra-tumoral lactate ratio and [^68^Ga]Ga-NODAGA-RGD distribution appears heterogeneous, without a clear spatial correspondence between [^68^Ga]Ga-NODAGA-RGD-PET and [1-^13^C]lactate to total ^13^C MRSI. Pyruvate is primarily seen in major vasculature (thin arrow, Fig. 4(1d)), and the cavity in the top region, presumably an encapsulated fluid collection due to a biopsy performed prior to the scanning session (thick arrow, Fig. 4(1d)).

The tumor of patient #2 (malignant melanoma) appears anatomically homogenous and generally shows very little tumor signal for both modalities, except some [1-^13^C]lactate to total ^13^C ratio in a few pixels. There is a relatively high uptake of [^68^Ga]Ga-NODAGA-RGD in the soft tissue of the paw (white arrows, Fig. 4(2b)).

The tumor of patient #3 (osteosarcoma) shows a heterogeneous [^68^Ga]Ga-NODAGA-RGD uptake around the central bone region (green outline). The [1-^13^C]lactate to total ^13^C ratio also appears spatially heterogeneous and shows some visual concurrence with the RGD distribution. Pyruvate is primarily seen in a major blood vessel along the edge of the tumor (white arrow, Fig. 4(3d)).

The tumor in patient #4 (thyroid carcinoma) shows a very heterogeneous distribution for both modalities, which corresponds with the follicular nature of the anatomical imaging. There appears to be no correlation between modalities, with a tendency of high [^68^Ga]Ga-NODAGA-RGD uptake at superficial tumor area, whereas high [1-^13^C]lactate to total ^13^C ratio occurs more centrally.

The subcutaneous tumor in patient #5 (soft tissue sarcoma) appears to have an area of central necrosis (white arrow, Fig. 4(5a)). [^68^Ga]Ga-NODAGA-RGD uptake is high in the vital tumor tissue, whereas [1-^13^C]lactate to total ^13^C ratio appears more evenly distributed.

The tumor in patient #6 (thyroid carcinoma) is highly vascularized with a large area of central necrosis (excluded from ROI, white star, Fig. 4(6a)). [^68^Ga]Ga-NODAGA-RGD and [1-^13^C]lactate to total ^13^C ratio are distributed heterogeneously in the tumor, with high spatial concurrence. Both [1-^13^C]pyruvate and [1-^13^C]lactate is primarily seen in the ventral tumor region (white arrow, Fig. 4(6d)), but overall, the ^13^C signal is potentially affected by the MR coil sensitivity profile.

The tumor in patient #7 (thyroid carcinoma) is clearly delineated, confined within the capsule and exhibits a necrotic core. Both [^68^Ga]Ga-NODAGA-RGD uptake and [1-^13^C]lactate to total ^13^C ratio is seen in the vital tumor tissue, the latter again influenced by the MR coil sensitivity profile.

### Intra-tumoral heterogeneity of [^68^Ga]Ga-NODAGA-RGD and [1-^13^C]lactate to total ^13^C ratio distribution are not related in general

The spatial relation of [^68^Ga]Ga-NODAGA-RGD uptake and [1-^13^C]lactate generation in tumors was further studied by a voxel-wise analysis, with results shown in Table 2. Voxels were only included if 90% of the voxel was inside the radiologist-delineated ROI. Tumors in patients #2 and #5 are omitted from further discussion due to poor ^13^C signal and small number of relevant voxels. Of the remaining five tumors, [^68^Ga]Ga-NODAGA-RGD uptake and [1-^13^C]lactate to total ^13^C ratio were weakly and non-significantly correlated in three, a significant positive correlation was observed for the tumor in patient #6, and a significant negative correlation was observed for the tumor in patient #7. A similar pattern was seen for the voxel-wise correlation of [^68^Ga]Ga-NODAGA-RGD uptake and [1-^13^C]lactate (not normalized to total ^13^C), with non-significant correlation in two tumors, a significant positive correlation in tumors of patients #3 and #6, and a significant negative correlation for the tumor in patient #4.

**Table 2:** Voxel-wise analysis of [^68^Ga]Ga-NODAGA-RGD-PET distribution and [1-^13^C] lactate to total ^13^C ratio for 7 patients

Patient	# voxels	(lac-RGD)			(ratio-RGD)			Comments
		*r*	*r*^2^	*p*	*r*	*r*^2^	*p*	
1	13	− 0.0867	0.00751	0.7783	0.1661	0.0276	0.5877	
2	14	0.5233	0.274	0.0548	0.5921	0.351	0.0257*	Poor signal quality
3	8	0.7562	0.571	0.0299*	0.0337	0.00114	0.9369	
4	11	− 0.628	0.394	0.0386*	0.1411	0.0199	0.6791	Negative lactate correlation
5	3	0.9836	0.967	0.1154	− 0.7598	0.577	0.4506	Too few voxels
6	39	0.5876	0.345	0.0001*	0.7345	0.539	0.0001*	
7	14	− 0.0893	0.00797	0.7615	− 0.5703	0.325	0.0332*	

### [1-^13^C]pyruvate metabolism showed no systematic relation with [^68^Ga]Ga-NODAGA-RGD-PET across tumors

**Fig. 5:**
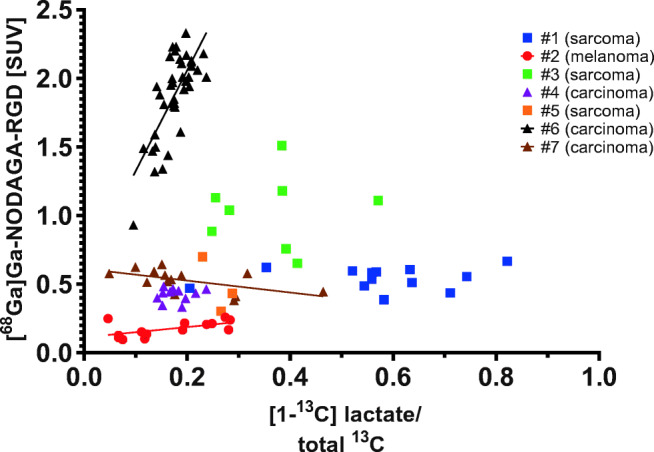
Plot showing [^68^Ga]Ga-NODAGA-RGD-PET uptake [SUV] versus [1-^13^C]lactate to total ^13^C ratio for all tumor voxels and all patients. Patient numbers (Table 1 and 2) are given in the legend. Overall tumor types are denoted by squares (sarcoma), triangles (carcinoma), and circles (melanoma). Lines are shown for within patient significant voxel-wise correlations

Figure 5 shows [^68^Ga]Ga-NODAGA-RGD-PET uptake [SUV (g/mL)] versus [1-^13^C]lactate to total ^13^C ratio in all tumor voxels for all patients. There is no apparent relation between angiogenesis and metabolism across tumors, as was also observed for the maximum values of RGD uptake and lactate ratio in Fig. 3. Please note that due to partial volume effects of the resampling of PET to ^13^C-MRSI voxels, RGD uptake values are systematically lower than in Fig. 2.

Lines are shown for within-tumor significant voxel-wise correlation of RGD uptake and ^13^C-lactate ratio. Both positive and negative correlations tend to appear for low values of the lactate ratio.

Within each patient tumor, the range of RGD uptake and ^13^C-lactate ratio is smaller, or much smaller, than the range of values across all patient tumors. Apparently, tumor voxel values of RGD uptake and [1-^13^C]lactate to total ^13^C ratio in Fig. 5 tend to group in clusters for each patient tumor. For tumors in patients #1, #2, #3, and #6, the clusters have small overlap between patients. Tumors in patients #4, #5, and #7 show some overlap of both RGD uptake and lactate ratio, with relatively small values of RGD uptake (approximately 0.3–0.7 SUV) and lactate ratio (approximately 0.1–0.3).

## Discussion

In the present work, we demonstrated the combined [^68^Ga]Ga-NODAGA-RGD-PET for angiogenesis and hyperpolarized [1-^13^C]pyruvate-MRSI for probing energy metabolism in a single session. Both RGD uptake and [1-^13^C]lactate generation showed a large degree of heterogeneity across tumor types and spatially within tumors. We found no obvious pattern of relation or consistent correlation between the two molecular imaging modalities. As illustrated in Fig. 5, the tumor voxel values of RGD uptake and lactate ratio for each canine cancer patient tends to aggregate in separated groups, indicating that [^68^Ga]Ga-NODAGA-RGD-PET and hyperpolarized [1-^13^C]pyruvate-MRSI may provide complementary cancer phenotypic information.

[^68^Ga]Ga-NODAGA-RGD-PET showed general tumor uptake in 10 out of 11 canine patients, with a varying degree of heterogeneity, indicating the disordered angiogenesis and vasculature present in cancer. Uptake values [SUV] varied across cancer types, although no pattern was seen in this regard. PET image contrast may potentially be affected by several factors, such as blood clearance, tumor vasculature, receptor dynamics, stability of the chelator, and radionuclide decay. Uptake values in the present study showed a significant, albeit small difference from 20 to 60 min uptake time, indicating robustness for clinical use. Most clinically applied RGD-based PET tracers to date have been based on ^18^F, and somewhat higher SUV values have been reported [29], but our findings are comparable with other reports of ^68^Ga-based RGD tracers [32, 40–42]. No adverse events were identified during or after the tracer injections. Visualizing the Warburg effect by hyperpolarized [1-^13^C]pyruvate-MRSI resulted in [1-^13^C]lactate generation in most tumors. Tumor type dependence confirmed the pattern previously reported by our group [17, 20], with sarcomas tending to exhibit higher [1-^13^C]lactate to total ^13^C ratio compared to carcinomas (Fig. 5).

Tumor uptake patterns of [^68^Ga]Ga-NODAGA-RGD and [1-^13^C]lactate did not correspond in general. Only two of five tumors showed a significant voxel-wise positive correlation of [^68^Ga]Ga-NODAGA-RGD uptake and [1-^13^C]lactate imaging and one of five showed a significant negative correlation. As well, when comparing [^68^Ga]Ga-NODAGA-RGD uptake with the [1-^13^C]lactate ratio, no clear picture of spatial correspondence emerged. In agreement with this quantitative correlation analysis (Table 2), the overall lack of similarity of uptake patterns is also qualitatively apparent in Fig. 3, underscoring individual tumor heterogeneity and phenotype. Previous hyperPET reports from our group [16, 17] showed an overall spatial correlation between ^13^C-lactate and [^18^F]FDG uptake in tumors; however, it should be noted that tumor types in the present study were not matched to those earlier study.

We presented the basic hypothesis that as hypoxia-induced transcription factors induce both angiogenesis and glycolysis, the imaging modalities reflecting these processes might be correlated. However, the Warburg effect is independent of hypoxia, and tumors may have both hypoxic and non-hypoxic regions. These factors may explain the lack of overall spatial correspondence of [^68^Ga]Ga-NODAGA-RGD uptake with [1-^13^C]lactate generation, found in this study. This is in line with findings in earlier studies of FDG-PET and RGD-tracers [32, 43].

As visualized in Fig. 5, tumor voxel values of [^68^Ga]Ga-NODAGA-RGD uptake and [1-^13^C]lactate to total ^13^C ratio tend to group in clusters for each canine cancer patient. Alternatively stated, the intra-tumoral range of [^68^Ga]Ga-NODAGA-RGD uptake and [1-^13^C]lactate to total ^13^C ratio is smaller than the range of values across all tumors. The clusters of each tumor show little overlap for at least four of seven tumors. It is noteworthy that this separation of tumors occurs only in the combined parameter space of [^68^Ga]Ga-NODAGA-RGD uptake and [1-^13^C]lactate to total ^13^C ratio. Tumor voxels from patient #1 have higher values of [1-^13^C]lactate to total ^13^C ratio than almost all other tumor voxels, but [^68^Ga]Ga-NODAGA-RGD uptake values are similar to tumor voxels from many other patients. Conversely, [^68^Ga]Ga-NODAGA-RGD uptake for patient #6 is higher than almost all other tumor voxels, but values for [1-^13^C]lactate to total ^13^C ratio are similar to tumor voxels from many other patients. For three of seven tumors shown in Fig. 5 (patients #4, #5, and #7), some overlap of both [^68^Ga]Ga-NODAGA-RGD uptake and [1-^13^C]lactate to total ^13^C ratio appears, with relatively small values of both modalities ([^68^Ga]Ga-NODAGA-RGD uptake approximately 0.3–0.7 SUV and lactate ratio approximately 0.1–0.3). These tumors appear to have follicular morphology, an area of central necrosis, and a necrotic colloid core, respectively. The tumor voxels with [^68^Ga]Ga-NODAGA-RGD uptake 0.3–0.7 SUV and lactate ratio 0.1–0.3 could then overall be expected to represent regions with a relatively low number of active tumor cells. A single voxel from patient #1 also has a [^68^Ga]Ga-NODAGA-RGD uptake and lactate ratio value in this overlap region and covers a possible biopsy cavity (Fig. 1).

The data of Fig. 5 may be interpreted as follows: The combined molecular imaging of angiogenesis and deregulated tumor cell energy metabolism appears to characterize distinct cancer phenotypes, which may be of great importance for clinical therapeutic decision making. Further studies are warranted to determine if this imaging paradigm can aid in histopathological tumor characterization.

Clinical translation of hyperpolarized [1-^13^C]pyruvate-MRSI into human patients is gaining momentum [44, 45]. Around the world, important experience is starting to emerge [46–48]. While most studies using hyperpolarized tracers are performed in a radiological (MR only) setting, we believe there lies an increased value of molecular imaging when combining with other modalities, such as PET. As we see indications of in this present study, the combination of modalities can provide additional information, making a combined examination more informative than if each were obtained separately. This is in line with other research groups, that are currently also pursuing the paradigm of complementary diagnostics in practice [16, 49].

The present study has some limitations. Imaging of canine cancer patients with spontaneous tumors is superior to cancer xenograft models in many regards, especially with respect to natural vasculature and intact immune system response. On the other hand, recruitment of this patient group limits the options of controlling treatment and cancer types, resulting in the mixed group of tumors. Even large animals must be anesthetized during the examination to avoid motion errors. Anesthesia can influence [1-^13^C]pyruvate-MRSI [50] and perhaps also [^68^Ga]Ga-NODAGA-RGD-PET, although less likely, as this passive interaction is receptor-mediated. This limitation should be taken into consideration during clinical translation.

The limited spatial resolution of both PET and in particular ^13^C MRSI implies that tumor heterogeneity on the millimeter scale is not captured. The importance of this in the context of therapeutic management is not known. As seen for patient #1 (Fig. 1), the small areas of necrosis visualized by anatomical MRI are hardly reflected in the spatial heterogeneity of the ^13^C MRSI. Still, the tumor heterogeneity of patient #4 (Fig. 4) is sufficiently reflected in both imaging modality measures to both visually and quantitatively conclude that uptake patterns of [^68^Ga]Ga-NODAGA-RGD and [1-^13^C]lactate to total ^13^C ratio do not correspond. Current technical developments of both PET and ^13^C MRSI acquisition schemes will improve image resolution [44].

The ^13^C metabolite maps are additionally affected by the MR surface coil profile, as seen for patient #6 (Fig. 4(6d)). This effect is mitigated by using the [1-^13^C]lactate to total ^13^C ratio as a measure, hence the focus on [1-^13^C]lactate to total ^13^C ratio for the comparisons to [^68^Ga]Ga-NODAGA-RGD uptake in the present work. Further development in acquisition hardware and sequences are needed to overcome this limitation. Finally, the interpretations of the combined imaging of energy metabolism and angiogenesis put forward here would benefit from postoperative histological verification as performed by Bachawal et al. [16].

In conclusion, we demonstrated that combined molecular imaging of angiogenesis by [^68^Ga]Ga-NODAGA-RGD-PET and deregulated tumor metabolism by [1-^13^C]pyruvate-MRSI is feasible in canine cancer patients with spontaneous tumors. RGD uptake and [1-^13^C]lactate generation had no overall correspondence, neither spatially nor across patients. As well, in combination (but not separately) of [^68^Ga]Ga-NODAGA-RGD-PET and [1-^13^C]pyruvate-MRSI showed a distinct clustering of tumor voxel values. Overall, the results suggest that [^68^Ga]Ga-NODAGA-RGD-PET and [1-^13^C]pyruvate-MRSI may provide complementary information indicating that the non-invasive hyperPET combined imaging of angiogenesis and glycolysis is able to aid in cancer phenotyping. This may lead to options for improved treatment by tailoring medication combinations to the individual patient.
